# Large deletions including inverted repeats of the pseudo*CHS* gene in seed-coat-pigmented mutants derived from Japanese yellow soybean cultivars

**DOI:** 10.1270/jsbbs.25021

**Published:** 2025-08-26

**Authors:** Mashiro Yuhazu, Yudai Higuchi, Akira Kanazawa, Mineo Senda

**Affiliations:** 1 Research Faculty of Agriculture, Hokkaido University, Sapporo, Hokkaido 060-8589, Japan; 2 Faculty of Agriculture and Life Science, Hirosaki University, Bunkyo, Hirosaki, Aomori 036-8561, Japan

**Keywords:** deletion, Enrei, genome, *GmIRCHS*, mutation, seed coat, soybean

## Abstract

In many yellow soybean (*Glycine max*) cultivars grown in Japan, pigmentation is suppressed throughout the seed coat, including the hilum region. This phenomenon is due to naturally occurring RNA silencing of chalcone synthase (*CHS*) genes, which is induced by the *I* allele of the *I* locus. A candidate for the *I* allele, designated *GmIRCHS*, includes inverted repeats of the pseudo*CHS* gene and is clustered with the *ICHS1* gene. Fully pigmented seeds are sometimes produced by yellow soybean cultivars as a result of a spontaneous mutation. The causal DNA changes are deletions that involve *GmIRCHS*. While small deletions of 0.8–3.3 kb have been previously characterized, the details for larger deletions remain unknown. Here, we characterized these large deletions using a recently released whole-genome sequence for the Japanese yellow soybean cultivar ‘Enrei’. The deletions encompass 17- to 38-kb DNA regions, which involve *GmIRCHS* and some or all of the neighboring genes *GmJ1*, *P450*, *Transferase*, and *ICHS1*. These data suggest that the DNA region surrounding *GmIRCHS* is prone to large structural changes, leading to reversion to pigmentation of the seed coat.

## Introduction

In soybean (*Glycine max* L.), the distribution of anthocyanins and proanthocyanidins in the seed coat is controlled by the *I* locus (Palmer *et al.* 2004, Senda *et al.* 2012, Todd and Vodkin 1993). Multiple genotypes in soybean are responsible for yellow seed phenotypes, which are distinguished by the presence or absence of pigmentation in the hilum region. The *I* allele inhibits pigmentation of the entire seed coat, including the hilum region (Bernard and Weiss 1973). The genotype of the *I* locus in yellow soybean cultivars with a nonpigmented hilum (hereafter yellow-hilum cultivars) is *II*. The *i^i^* allele inhibits pigmentation in the seed coat, but permits pigmentation only in the hilum region (Bernard and Weiss 1973); thus, the genotype of the *I* locus in yellow soybean cultivars with a pigmented hilum (hereafter pigmented-hilum cultivars) is *i^i^i^i^*. The *i* allele permits pigmentation of the entire seed coat, including the hilum region, and thus pigmented soybean cultivars have the *ii* genotype (Bernard and Weiss 1973). Among the seeds harvested from yellow soybean cultivars, pigmented seeds have been infrequently observed and are the result of a spontaneous mutation from either *I* or *i^i^* to *i* (Todd and Vodkin 1996). The yellow seed phenotype is the agronomically preferred trait in yellow soybean cultivars. Therefore, although the percentage is usually low, the seed-coat-pigmented mutations (hereafter scp mutations) are one of the causes for concern among farmers and seed suppliers (Bernard and Weiss 1973).

In yellow soybean cultivars with the *II* (yellow-hilum cultivars) or *i^i^i^i^* (pigmented-hilum cultivars) genotype, inhibition of seed coat pigmentation is caused by naturally occurring RNA silencing of chalcone synthase (*CHS*) genes, which is termed *CHS* silencing (Senda *et al.* 2004, Tuteja *et al.* 2004). Sequence analysis of a BAC clone harboring the *I* locus from the pigmented-hilum cultivar ‘Williams 82’ (Wm82) with the *i^i^i^i^* genotype revealed a candidate region for the *i^i^* allele: an inverted repeat arrangement of an identical *CHS1–CHS3–CHS4* cluster of 10.91 kb separated by a 5.71-kb intervening sequence (Clough *et al.* 2004). This candidate region, referred to as *CHS1-3-4-Hypo-CHS4-3-1*, is located on chromosome 8 (Gm08) (Cho *et al.* 2019) (Supplemental Fig. 1). Two different models for the induction of *CHS* silencing by the *i^i^* candidate have been proposed, but the details remain unclear (Clough *et al.* 2004, Jia *et al.* 2020, Xie *et al.* 2019).

We identified a candidate region for the *I* allele, designated *Glycine max* inverted repeat of pseudo*CHS* gene (*GmIRCHS*), in the genome of the Japanese yellow-hilum cultivar ‘Toyohomare’ with the *II* genotype (Kasai *et al.* 2007). *GmIRCHS* is situated 680 bp upstream of a *CHS1* gene, designated *ICHS1*, which together form a cluster, *GmIRCHS–ICHS1* (Kasai *et al.* 2007, Senda *et al.* 2002a). *GmIRCHS* is composed of a 5ʹ-portion, including the promoter region, of a DnaJ gene named *GmJ1*, and a very closely spaced (78-bp distance) and tail-to-tail-arranged 1,087-bp inverted repeat of pseudo*CHS3* (Kasai *et al.* 2007) (Supplemental Fig. 2). This chimeric structure indicates that *GmIRCHS* could be transcribed and its transcript likely forms the double-stranded RNA (dsRNA) structure of the *CHS* gene (*CHS* dsRNA) triggering *CHS* silencing (Kasai *et al.* 2007). Indeed, *CHS* dsRNAs derived from *GmIRCHS* have been detected in the seed coat of a Japanese yellow-hilum breeding line (‘Karikei 557’) with the *II* genotype (Kurauchi *et al.* 2011). These results led us to propose a model of *CHS* silencing by *GmIRCHS* in which *GmIRCHS* transcripts serve to induce extensive degradation of other *CHS* transcripts (Senda *et al.* 2012). The mutation from *I* to *i* was associated with deletions, including *GmIRCHS* in full or in part (Kasai *et al.* 2007, Senda *et al.* 2002b, 2013). Furthermore, the 5ʹ- and 3ʹ-ends of the deletion regions were identified in the scp mutants (harboring the *ii* genotype) derived from many Japanese yellow-hilum cultivars and breeding lines (harboring the *II* genotype). These scp mutants were categorized into two types: in type II scp mutants, small regions of several kb were deleted within the *GmIRCHS–ICHS1* cluster, whereas in type I scp mutants, the 5ʹ-ends of the deletion regions were located in an internal position or flanking regions of a cytochrome P450 gene (hereafter simply *P450*) and the 3ʹ-ends of the deletion regions were located in the *GmIRCHS–ICHS1* cluster (Senda *et al.* 2013). Unlike the type II scp mutants, in the type I mutants the deleted sequences could not be determined in full because the *GmIRCHS–ICHS1* cluster was not continuously connected to the *P450* region in the available genome sequence of a Japanese yellow-hilum soybean. Among Japanese yellow-hilum soybean cultivars, a version 2 of the whole-genome sequence of ‘Enrei’ has been reported. However, the authors detected only one *CHS* cluster on chromosome 8 and did not identify the *GmIRCHS–ICHS1* cluster (Shimomura *et al.* 2015). ‘Enrei’ is a model cultivar for soybean genome research in Japan and possesses the *II* genotype. Recently, an updated version of the whole-genome sequence of ‘Enrei’ (the Enrei ver. 3.31 genome assembly), which was generated by Oxford Nanopore long-read sequencing, has been released (Yano *et al.* 2025). In the current study, first, we identified the genome position of the *GmIRCHS–ICHS1* cluster in the ‘Enrei’ reference genome (the Enrei ver. 3.31 genome assembly) at approximately 8,640 kb on chromosome 8 (chr08) and further identified three genes (*GmJ1*, *P450*, and a transferase gene) located in the 5ʹ-upstream region from the *GmIRCHS–ICHS1* cluster. Second, we identified the large deletion regions in type I scp mutants derived from ‘Enrei’ by performing an *in silico* analysis. Recently, we determined that an scp mutant derived from the Japanese yellow-hilum cultivar ‘Suzuyutaka’, designated SYM, belonged to the type I group (Yuhazu *et al.* 2024). Accordingly, we identified a region deleted in SYM based on the ‘Enrei’ genome sequence, in addition to those regions deleted in ‘Enrei’ type I scp mutants. Through these analyses, we aimed to gain a detailed understanding of the DNA structural changes that occurred during the reversion to pigmented seed coats from nonpigmented seed coats generated by *CHS* silencing.

## Materials and Methods

### Plant materials

**Fig. 1.**
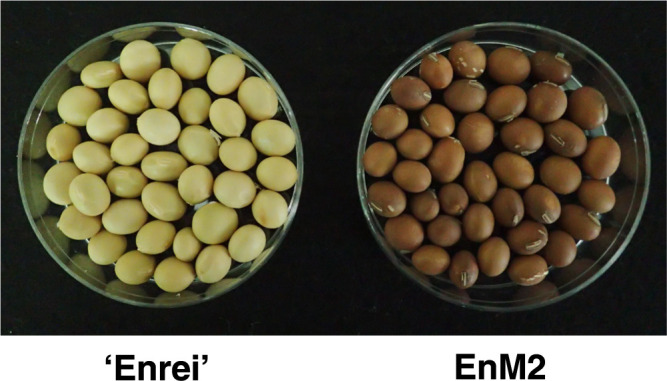
Seeds of a yellow soybean cultivar, ‘Enrei’ with the *II* genotype, and its seed-coat-pigmented mutant (EnM2) with the *ii* genotype. Seeds of scp mutants exhibit pigmentation of the entire seed coat, including the hilum region.

‘Enrei’ is a model cultivar for soybean genome research in Japan. Seven independent scp mutants (EnM1–EnM7) derived from ‘Enrei’ were collected in different fields in Niigata Prefecture, Japan. However, only in EnM1, the 5ʹ- and 3ʹ-ends of the deletion region could not be determined; therefore, EnM1 was excluded from our previous study (Senda *et al.* 2013). Among the remaining six scp mutants (EnM2–EnM7), four scp mutants (EnM2, EnM3, EnM5, and EnM6) classified in the type I group (Senda *et al.* 2013) were tested in the present study. SYM was isolated from the M_2_ progeny of a population of cultivar ‘Suzuyutaka’ mutagenized with 20 kR (5.16 C/kg) X-ray irradiation at the Shonai Regional Center for Biotechnology, Yamagata Prefecture, Japan (Yuhazu *et al.* 2024). Both Japanese yellow-hilum cultivars (‘Enrei’ and ‘Suzuyutaka’) possess the *II* genotype, whereas EnM2, EnM3, EnM5, EnM6, and SYM have the *ii* genotype of the *I* locus (Senda *et al.* 2013, Yuhazu *et al.* 2024). In common, seeds of scp mutants exhibit pigmentation of the entire seed coat, including the hilum region (Kasai *et al.* 2007, Senda *et al.* 2004, Yuhazu *et al.* 2024) (Fig. 1).

### Sequence data analysis

The genome sequence of ‘Enrei’ (the Enrei ver. 3.31 genome assembly) was accessed with the JBrowse2 genome browser of the Daizu-net portal (https://daizu-net.dna.affrc.go.jp/ap/top). Sequences of genes located 5ʹ-upstream from *CHS1-3-4-Hypo-CHS4-3-1* were searched in the Wm82.a4.v1 genome assembly available from Phytozome v13 (https://phytozome-next.jgi.doe.gov). The sequence of the *GmIRCHS–ICHS1* cluster was obtained from DDBJ/EMBL/GenBank under the accession number AB264311 (Kasai *et al.* 2007). Nucleotide sequences of the DNA regions adjacent to the deleted regions in the type I scp mutants were used for comparison with the reference ‘Enrei’ genome sequence. The DNA regions in ‘Enrei’ type I scp mutants were amplified by inverse PCR and their nucleotide sequences were analyzed by the dideoxy chain termination method (Senda *et al.* 2013). The nucleotide sequences in EnM2, EnM3, EnM5, and EnM6 were submitted to DDBJ/EMBL/GenBank under the accession numbers AB822566, AB822567, AB822569, and AB822570, respectively. Similarly, the DNA regions adjacent to the deleted region in SYM were identified by analyzing a PCR-amplified fragment (Yuhazu *et al.* 2024). The nucleotide sequence of the PCR-amplified fragment encompassing the deletion point in SYM was submitted to DDBJ/EMBL/GenBank under the accession number LC871491. Homologous sequences in the ‘Enrei’ genome were searched with the Basic Local Alignment Search Tool (BLAST) at Daizu-net (https://daizu-net.dna.affrc.go.jp/ap/bls).

## Results

### Identification of the *GmIRCHS–ICHS1* cluster and genes in its upstream region in the Enrei genome

A whole-genome sequence of soybean was first reported for a pigmented-hilum cultivar, ‘Wm82’, with the *i^i^i^i^* genotype (Schmutz *et al.* 2010). The *i^i^* candidate, *CHS1-3-4-Hypo-CHS4-3-1*, was mapped to a region on Gm08 from approximately 8,501 kb to 8,527 kb ‘Wm82’ genome position on Gm08 in the Wm82.a4.v1 assembly (Supplemental Fig. 1). Previous analyses of type I scp mutants have shown that the 5ʹ-ends of the deletion regions were located in the *P450* internal or flanking regions (Senda *et al.* 2013), as mentioned earlier. Considering the possibility that *P450* may be located in the region upstream of *CHS1-3-4-Hypo-CHS4-3-1*, we surveyed the ‘Wm82’ genome and determined that the *P450* gene (Glyma.08G109900) was present approximately 64-kb upstream of *CHS1-3-4-Hypo-CHS4-3-1* in the ‘Wm82’ genome (Supplemental Fig. 1). In ‘Wm82’, the *GmJ1* gene (Glyma.08G109700) and a transferase gene (Glyma.08G110000), hereafter simply *Transferase*, were located upstream and downstream of *P450*, respectively (Supplemental Fig. 1). Thus, in the ‘Wm82’ genome, three genes (*GmJ1*, *P450*, and *Transferase*) were located upstream from the *i^i^* allele.

**Fig. 2.**
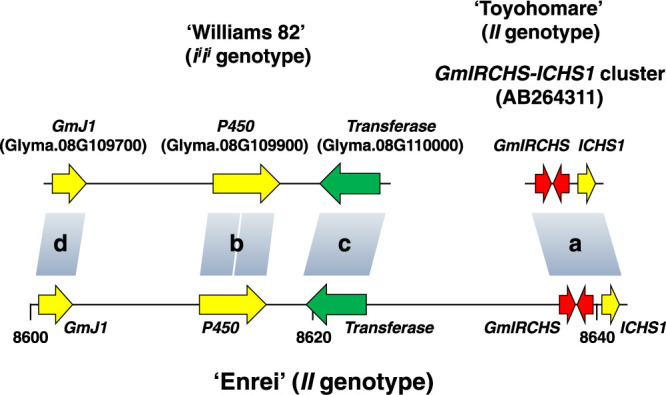
A diagram for mapping the *GmIRCHS–ICHS1* cluster and three genes in the ‘Enrei’ reference genome (the Enrei ver. 3.31 genome assembly) at the approximately 8,600 to 8,640 kb positions on chr08: (a) *GmIRCHS–ICHS1* cluster, (b) a cytochrome P450 gene (*P450*), (c) a transferase gene (*Transferase*), and (d) a DnaJ gene (*GmJ1*). Horizontal arrows indicate genes and direction: yellow horizontal arrows indicate genes on the sense strand, a green horizontal arrow indicates a gene on the opposite strand, and red horizontal arrows indicate *GmIRCHS*.

Next, using BLAST on the Daizu-net platform, we searched for the homologous sequence of the *GmIRCHS–ICHS1* cluster (AB264311) in the genome of ‘Enrei’ with the *II* genotype. A perfectly matched sequence was detected at the genome position on chr08 around 8,640 kb (8,636,590–8,642,946) (Fig. 2, Supplemental Fig. 2). As a result of the BLAST search for the *P450* sequence (Glyma.08G109900), we observed that the *P450* gene was located approximately 20 kb upstream of the *GmIRCHS–ICHS1* cluster in the ‘Enrei’ genome (Fig. 2). Unexpectedly, BLAST detected two separate regions that were highly homologous to Glyma.08G109900 (*P450*) near the approximately 8,610-kb ‘Enrei’ genome position (Fig. 2): 8,611,931–8,614,840 (98% nucleotide identity, 36-bp gaps, Supplemental Fig. 3A) and 8,614,955–8,617,097 (99% nucleotide identity, 13-bp gaps, Supplemental Fig. 3B). This finding suggested that non-conserved sequences may be present between these two regions. The nucleotide sequence from positions 8,614,841 to 8,614,954 of the ‘Enrei’ genome was 5ʹ-GGCCCATAATATGTCAAAATGCTCGAAATTGAATGAAGAATCCCTTGGTTGGTCAACTCATTTTCCCAAAAAATGCCTCTAAGTAACTCAAAAATAGGATTTAAGATTAAAAAA-3ʹ, whereas the corresponding sequence of the ‘Wm82’ genome was 5ʹ-CCGCCGCCTTACTAAGTACGCCTATTTCGGTTATT(N_100_)CG-3ʹ, indicating that these two sequences were different. Both sequences exist within an intron and may be derived from different origins. Furthermore, detailed sequence comparisons between ‘Enrei’ and ‘Wm82’ indicated that a sequence perfectly matching that of Glyma.08G110000 (*Transferase*) was present on the opposite strand at the ‘Enrei’ genome position of approximately 8,620 kb (8,623,430–8,618,956) (Fig. 2, Supplemental Fig. 4). At the ‘Enrei’ genome position of approximately 8,600 kb (8,600,534–8,603,140), although there were 37-bp gaps, a sequence highly homologous (99% nucleotide identity) to that of Glyma.08G109700 (*GmJ1*) was located (Fig. 2, Supplemental Fig. 5). Thus, the distribution of these three genes (*GmJ1*, *P450*, and *Transferase*) was conserved between ‘Wm82’ and ‘Enrei’ (Fig. 2).

### Type I scp mutations are large deletions including *GmIRCHS*

**Fig. 3.**
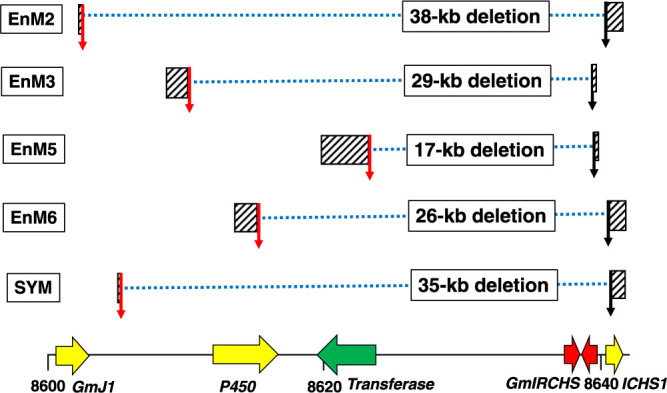
Deletion regions and their sizes in type I scp mutants. Positions of the 5ʹ- and 3ʹ-ends of the deletion regions are denoted by vertical red and black arrows, respectively. Hatched boxes indicate regions of the analyzed sequences in the type I scp mutants. Blue dotted lines indicate the deleted regions. *GmIRCHS–ICHS1* cluster and its 5ʹ-upstream region is shown at the approximately 8,600 to 8,640 kb positions on chr08 of the ‘Enrei’ reference genome (the Enrei ver. 3.31 genome assembly). The size of the deleted region in SYM was estimated based on the assumption that the genomic structure of ‘Suzuyutaka’ is identical to that of ‘Enrei’ at this locus.

In our previous study, we identified the 5ʹ-ends of the deletion regions in type I scp mutants of ‘Enrei’, comprising EnM2, EnM3, EnM5, and EnM6, by comparison with the *P450* sequence and its flanking sequences, and further identified the 3ʹ-ends of the deletion regions by comparison with the *GmIRCHS–ICHS1* sequence (Senda *et al.* 2013). In the present study, using the Daizu-net BLAST, the nucleotide sequences of EnM2 (AB822566), EnM3 (AB822567), EnM5 (AB822569), and EnM6 (AB822570) were each compared with the ‘Enrei’ reference genome (the Enrei ver. 3.31 genome assembly). We mapped the 5ʹ- and 3ʹ-end points on the ‘Enrei’ genome sequence and determined the areas between the 5ʹ- and 3ʹ-end points as deletion regions (Fig. 3). Large deletions that varied in size from approximately 17 kb (EnM5) to 38 kb (EnM2), occurred in ‘Enrei’ type I scp mutants (Fig. 3, Supplemental Figs. 6–9). Although deletions of *GmIRCHS* were common in type I scp mutants, an additional four genes (*GmJ1*, *P450*, *Transferase*, and *ICHS1* in EnM2) (Supplemental Fig. 6), three genes (*P450*, *Transferase*, and *ICHS1* in EnM6) (Supplemental Fig. 9), two genes (*P450* and *Transferase* in EnM3) (Supplemental Fig. 7), or one gene (*Transferase* in EnM5) (Supplemental Fig. 8) were also deleted (Fig. 3). Similar to ‘Enrei’ type I scp mutants, by comparing the nucleotide sequence of SYM (LC871491) with the ‘Enrei’ genome sequence, deletion of an approximately 35-kb region including *P450*, *Transferase*, *GmIRCHS*, and *ICHS1* was estimated in SYM, on the assumption that the genomic structure of ‘Suzuyutaka’ is identical to that of ‘Enrei’ at this locus (Fig. 3, Supplemental Fig. 10).

## Discussion

Deletion regions in the *I* allele have previously been classified into types I and II, but the entire deletion region in type I has not been identified (Senda *et al.* 2013). In the present study, we aimed to identify the deletion regions in several ‘Enrei’ scp mutants (EnM2, EnM3, EnM5, and EnM6) and a ‘Suzuyutaka’ scp mutant (SYM) classified as type I, based on the genome sequence of ‘Enrei’ with the *II* genotype. First, we investigated the location of the *GmIRCHS–ICHS1* cluster on chr08 in the ‘Enrei’ genome and established that it is located at the genome position of approximately 8,640 kb (Fig. 2). Next, a transferase gene (*Transferase*) (Glyma.08G110000) was located on the opposite strand approximately 14 kb upstream from the *GmIRCHS–ICHS1* cluster (Fig. 2). A cytochrome P450 gene (*P450*) (Glyma.08G109900) was located approximately 20 kb upstream from the *GmIRCHS–ICHS1* cluster, and *GmJ1* (Glyma.08G109700) was located approximately 9 kb upstream from *P450* (Fig. 2). The 5ʹ- and 3ʹ-ends of the deletion regions in type I ‘Enrei’ scp mutants were mapped and subsequently the deletion regions were identified (Fig. 3). In this manner, it was revealed that EnM2 had a deletion of 38 kb (Supplemental Fig. 6), EnM3 had a deletion of 29 kb (Supplemental Fig. 7), EnM5 had a deletion of 17 kb (Supplemental Fig. 8), and EnM6 had a deletion of 26 kb (Supplemental Fig. 9). Similarly, a deletion in SYM was estimated to be 35 kb based on the genome sequence of ‘Enrei’ (Fig. 3, Supplemental Fig. 10). Taking into account the DNA structural changes in ‘Enrei’ scp mutants classified as type II (EnM4: deletion of 3.3 kb in the *GmIRCHS–ICHS1* cluster; and EnM7: deletion of 0.8 kb in *GmIRCHS*) (Senda *et al.* 2013), it was revealed that the mutation from *I* to *i* was caused by a deletion. The only difference between types I and II is likely the size of the deletion. A notable common feature was that all of these types I and II scp mutants, which were derived from the *I*→*i* mutation, had a deletion of *GmIRCHS* in full or in part, supporting the notion that *GmIRCHS* is the *I* allele (Kasai *et al.* 2007). Why is *GmIRCHS* prone to deletion? In a previous analysis of SYM, we noted that the 5ʹ- and 3ʹ-end regions of the deletion region can form secondary structures and are AT-rich, which may be less stable in the DNA structure (Yuhazu *et al.* 2024), suggesting that deletions in the *GmIRCHS* region may occur to abrogate genomic instability and vulnerability of rearrangements. In addition, segmental duplication and insertion have occurred in this chromosomal region during evolution (Cho *et al.* 2019), which implies that this region may be prone to structural changes. In the future, epigenetic research, including investigation of chromatin structures, will be important to elucidate the deletion-prone mechanisms in the *GmIRCHS* region, which may ultimately lead to effective inhibition of seed coat pigmentation.

## Author Contribution Statement

MS designed the research. MS and AK wrote the manuscript. MY, YH, AK, and MS analyzed the sequence data. All authors read and approved the manuscript.

## Supplementary Material

Supplemental Figures
