# Supplementary material for: Large deletions including inverted repeats of the pseudoCHS gene in seed-coat-pigmented mutants derived from Japanese yellow soybean cultivars
Source: Breed Sci. 2025 Aug 26;75(4):334–8. doi: 10.1270/jsbbs.25021 (PMC13051633; doi:10.1270/jsbbs.25021)
Supplement: Supplementary file 1 — Supplemental Figures [file 75_334_s1.pdf]

doi: 10.1270/jsbbs.25021

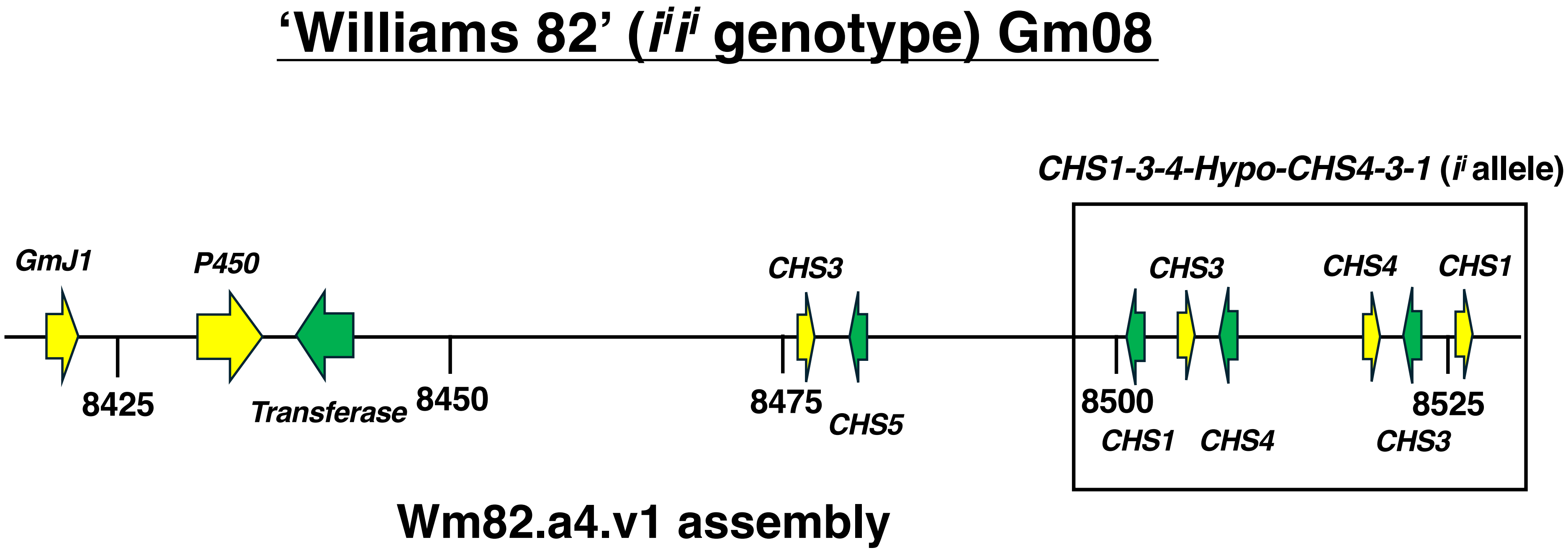

Supplemental Fig. 1. Organization of the reference genome for soybean 'Williams 82' with the $i^i i^i$ genotype at the approximately 8,425 to 8,525 kb genome positions on chromosome 8 (Gm08). This map was created based on the results obtained with the genome browser of Phytozome v13. Gene annotation was provided by Phytozome v13 and Cho *et al*. (2019). Horizontal arrows indicate genes and direction: yellow horizontal arrows indicate genes on the sense strand, and green horizontal arrows indicate genes on the opposite strand. Multiple *CHS* genes and three genes (*GmJ1*, *P450*, and *Transferase*) are presented. The region of an i^i candidate (*CHS1-3-4-Hypo-CHS4-3-1*) is indicated by a box.

```
1        GAATTCACCATCTCTTTTCACTTGTTTCTTAAGTTTGTTTGGATAATTTTTAAAAAAATACTTATAAAATAATAAAATAAGAATAAAAAATGAGATAATTTTTTCTATAAATTAAAATA  120
         ||||||||||||||||||||||||||||||||||||||||||||||||||||||||||||||||||||||||||||||||||||||||||||||||||||||||||||||||||||||||
8636590  GAATTCACCATCTCTTTTCACTTGTTTCTTAAGTTTGTTTGGATAATTTTTAAAAAAATACTTATAAAATAATAAAATAAGAATAAAAAATGAGATAATTTTTTCTATAAATTAAAATA  8636709

121      AAATTTCTATTAATTAATTTGTAAAACTCTCTAAAAAAATTAAAAATTAGCTAATAAATAACTCATTATGGAGAATTTTATGTAATTTTTTTTCTTTTTATTTTCTTTTCTGATAGGTT  240
         ||||||||||||||||||||||||||||||||||||||||||||||||||||||||||||||||||||||||||||||||||||||||||||||||||||||||||||||||||||||||
8636710  AAATTTCTATTAATTAATTTGTAAAACTCTCTAAAAAAATTAAAAATTAGCTAATAAATAACTCATTATGGAGAATTTTATGTAATTTTTTTTCTTTTTATTTTCTTTTCTGATAGGTT  8636829

241      CTTTGAGATAAACTTGCCTAGATAGACCCAAAAATGTAGATTTGTAAAACCCACAACCCAATGATACAAAGTCCATGCCTTGGCCTCCCACTCGTTTACTTATGGCTAAAACGACAGCTA  360
         ||||||||||||||||||||||||||||||||||||||||||||||||||||||||||||||||||||||||||||||||||||||||||||||||||||||||||||||||||||||||
8636830  CTTTGAGATAAACTTGCCTAGATAGACCCAAAAATGTAGATTTGTAAAACCCACAACCCAATGATACAAAGTCCATGCCTTGGCCTCCCACTCGTTTACTTATGGCTAAAACGACAGCTA  8636949

361      ATCACGCAAAACTAAATAAATGAAATGAGAACCATATAAAAAGAAAAAGTCATAAAGAGTAATAATAGCAACACACGCAAACCAAATCAAGTAAGAGCGAGAGTGTGATAGAGAGATAAT  480
         ||||||||||||||||||||||||||||||||||||||||||||||||||||||||||||||||||||||||||||||||||||||||||||||||||||||||||||||||||||||||
8636950  ATCACGCAAAACTAAATAAATGAAATGAGAACCATATAAAAAGAAAAAGTCATAAAGAGTAATAATAGCAACACACGCAAACCAAATCAAGTAAGAGCGAGAGTGTGATAGAGAGATAAT  8637069

481      GCGTTCTTATGGCTTAACCGTACCGGGAAGCGACCACCGCTTCTGCGTGCCCACCGTATCTCAGGTGTTCTTTAGGCCCAACAGGCCCATTAACCGAATCGCGTTTCCCAAGGCCACCCT  600
         ||||||||||||||||||||||||||||||||||||||||||||||||||||||||||||||||||||||||||||||||||||||||||||||||||||||||||||||||||||||||
8637070  GCGTTCTTATGGCTTAACCGTACCGGGAAGCGACCACCGCTTCTGCGTGCCCACCGTATCTCAGGTGTTCTTTAGGCCCAACAGGCCCATTAACCGAATCGCGTTTCCCAAGGCCACCCT  8637189

601      CAAGAACGACGGCTTCGTGGTGGACCTGAGCTTCTACGACCTTCTGGGAATACCGGAATCCGGTTCGGTAACGGAGATCAAGAACGCCTACAAGCAACTCGCCCGAAAGTACCACCCGGA  720
         ||||||||||||||||||||||||||||||||||||||||||||||||||||||||||||||||||||||||||||||||||||||||||||||||||||||||||||||||||||||||
8637190  CAAGAACGACGGCTTCGTGGTGGACCTGAGCTTCTACGACCTTCTGGGAATACCGGAATCCGGTTCGGTAACGGAGATCAAGAACGCCTACAAGCAACTCGCCCGAAAGTACCACCCGGA  8637309

721      CGTGTCGCCTCCGGGTCGGGTCGAGGAGTACACAAAGAGGTTTATTCAGGTGCAGGAGGCCTACGAAACCCTGTCTGATCCTTCCAGAAGAGCTATGTACGACAAGGACCTTAAACGAAG  840
         ||||||||||||||||||||||||||||||||||||||||||||||||||||||||||||||||||||||||||||||||||||||||||||||||||||||||||||||||||||||||
8637310  CGTGTCGCCTCCGGGTCGGGTCGAGGAGTACACAAAGAGGTTTATTCAGGTGCAGGAGGCCTACGAAACCCTGTCTGATCCTTCCAGAAGAGCTATGTACGACAAGGACCTTAAACGAAG  8637429

841      AGATCCTGAAGGAGAATCCCAGTGTTTGTGCATATATGGCACCTTCGTTGGATGCAAGGCAAGACATGGTGGTTGTGGAGGTACCAAAGTTGGGAAAAGAGGCTGCAACTAAGGCAATCA  960
         ||||||||||||||||||||||||||||||||||||||||||||||||||||||||||||||||||||||||||||||||||||||||||||||||||||||||||||||||||||||||
8637430  AGATCCTGAAGGAGAATCCCAGTGTTTGTGCATATATGGCACCTTCGTTGGATGCAAGGCAAGACATGGTGGTTGTGGAGGTACCAAAGTTGGGAAAAGAGGCTGCAACTAAGGCAATCA  8637549

961      AGGAATGGGGTCAACCCAAGTCCAAGATTACCCATCTCATCTTTTGCACCACTAGTGGTGTCGACATGCCTGGTGCTGATTATCAGCTCACTAAACTATTAGGCCTTCGTCCCTCCGTCA  1080
         ||||||||||||||||||||||||||||||||||||||||||||||||||||||||||||||||||||||||||||||||||||||||||||||||||||||||||||||||||||||||
8637550  AGGAATGGGGTCAACCCAAGTCCAAGATTACCCATCTCATCTTTTGCACCACTAGTGGTGTCGACATGCCTGGTGCTGATTATCAGCTCACTAAACTATTAGGCCTTCGTCCCTCCGTCA  8637669

1081     AGCGTTACATGATGTACCAACAAGGCTGCTTTGCCGGTGGCACGGTGCTTCGTTTGGCCAAAGACCTCGCTGAAAACAACAAGGGTGCTCGCGTGCTTGTCGTTTGTTCTGAGATCACCG  1200
         ||||||||||||||||||||||||||||||||||||||||||||||||||||||||||||||||||||||||||||||||||||||||||||||||||||||||||||||||||||||||
8637670  AGCGTTACATGATGTACCAACAAGGCTGCTTTGCCGGTGGCACGGTGCTTCGTTTGGCCAAAGACCTCGCTGAAAACAACAAGGGTGCTCGCGTGCTTGTCGTTTGTTCTGAGATCACCG  8637789

1201     CAGTCACATTCCGTGGCCCAACTGACACCCATCTTGATAGCCTTGTGGGTCAAGCCTTGTTTGGAGATGGTGCAGCCGCTGTCATTGTTGGATCAGACCCCTTACCAGTTGAAAAGCCTT  1320
         ||||||||||||||||||||||||||||||||||||||||||||||||||||||||||||||||||||||||||||||||||||||||||||||||||||||||||||||||||||||||
8637790  CAGTCACATTCCGTGGCCCAACTGACACCCATCTTGATAGCCTTGTGGGTCAAGCCTTGTTTGGAGATGGTGCAGCCGCTGTCATTGTTGGATCAGACCCCTTACCAGTTGAAAAGCCTT  8637909

1321     TGTTTCAGCTTGTCTGGACTGCCCAGACAATCCTTCCAGACAGTGAAGGGGCTATTGATGGACACCTTCGCGAAGTTGGTCTCACTTTCCATCTCCTCAAGGATGTTCCTGGACTCATCT  1440
         ||||||||||||||||||||||||||||||||||||||||||||||||||||||||||||||||||||||||||||||||||||||||||||||||||||||||||||||||||||||||
8637910  TGTTTCAGCTTGTCTGGACTGCCCAGACAATCCTTCCAGACAGTGAAGGGGCTATTGATGGACACCTTCGCGAAGTTGGTCTCACTTTCCATCTCCTCAAGGATGTTCCTGGACTCATCT  8638029

1441     CCAAGAATATTGAGAAGGCCTTGGTTGAAGCCTTCCAACCCTTGGGAATCTCCGATTACAATTCTATCTTCTGGATTGCACACCCTGGTGGACCCGCAATTTTGGACCAAGTGGAGGCTA  1560
         ||||||||||||||||||||||||||||||||||||||||||||||||||||||||||||||||||||||||||||||||||||||||||||||||||||||||||||||||||||||||
8638030  CCAAGAATATTGAGAAGGCCTTGGTTGAAGCCTTCCAACCCTTGGGAATCTCCGATTACAATTCTATCTTCTGGATTGCACACCCTGGTGGACCCGCAATTTTGGACCAAGTGGAGGCTA  8638149

1561     AGTTAGGCTTGAAGCCTGAAAAAATGGAAGCTACTAGGCATGTGCTCAGCGAGTATGGTAACATGTCAAGTGCATGTGTGCTATTCATCTTGGATCAAATGAGGAGGAAGTCATTAGAAA  1680
         ||||||||||||||||||||||||||||||||||||||||||||||||||||||||||||||||||||||||||||||||||||||||||||||||||||||||||||||||||||||||
8638150  AGTTAGGCTTGAAGCCTGAAAAAATGGAAGCTACTAGGCATGTGCTCAGCGAGTATGGTAACATGTCAAGTGCATGTGTGCTATTCATCTTGGATCAAATGAGGAGGAAGTCATTAGAAA  8638269

1681     ATGGACTTGGCACAACAGGTGAAGGCCTTGAATGGGGTGTGCTGTTTGGTTTTGGCCCTGGACTCACTGTTGAGACTGTTGTGCTCCGCAGTGTCACTGTCTAATCCATATATCTTGTGC  1800
         ||||||||||||||||||||||||||||||||||||||||||||||||||||||||||||||||||||||||||||||||||||||||||||||||||||||||||||||||||||||||
8638270  ATGGACTTGGCACAACAGGTGAAGGCCTTGAATGGGGTGTGCTGTTTGGTTTTGGCCCTGGACTCACTGTTGAGACTGTTGTGCTCCGCAGTGTCACTGTCTAATCCATATATCTTGTGC  8638389

1801     AAGACCAAGTCCTTTTCTTTCTTATTCATTCTTTCATGTTGAGTTTGAAAAATGTATTCTTTCTCTTCCTTTTTTTCCCTGCTTCCCTCACATGCTTCTTTTTTTATACCAGTAAATAAG  1920
         ||||||||||||||||||||||||||||||||||||||||||||||||||||||||||||||||||||||||||||||||||||||||||||||||||||||||||||||||||||||||
8638390  AAGACCAAGTCCTTTTCTTTCTTATTCATTCTTTCATGTTGAGTTTGAAAAATGTATTCTTTCTCTTCCTTTTTTTCCCTGCTTCCCTCACATGCTTCTTTTTTTATACCAGTAAATAAG  8638509

1921     AACATGTTATATTACTATTAATGCAATTAAGTTTTCACAAAATTCATCTATATATTCATGTAAATAAGAACATGTTTACTGGTATAAAAAAAGAAGCATGTGAGGGAAGCAGGGAAAAAA  2040
         ||||||||||||||||||||||||||||||||||||||||||||||||||||||||||||||||||||||||||||||||||||||||||||||||||||||||||||||||||||||||
8638510  AACATGTTATATTACTATTAATGCAATTAAGTTTTCACAAAATTCATCTATATATTCATGTAAATAAGAACATGTTTACTGGTATAAAAAAAGAAGCATGTGAGGGAAGCAGGGAAAAAA  8638629

2041     AGGAAGAGAAAGAATACATTTTTCAAACTCAACATGAAAGAATGAATAAGAAAGAAAAGGACTTGGTCTTGCACAAGATATATGGATTAGACAGTGACACTGCGGAGCACAACAGTCTCA  2160
         ||||||||||||||||||||||||||||||||||||||||||||||||||||||||||||||||||||||||||||||||||||||||||||||||||||||||||||||||||||||||
8638630  AGGAAGAGAAAGAATACATTTTTCAAACTCAACATGAAAGAATGAATAAGAAAGAAAAGGACTTGGTCTTGCACAAGATATATGGATTAGACAGTGACACTGCGGAGCACAACAGTCTCA  8638749

2161     ACAGTGAGTCCAGGGCCAAAACCAAACAGCACACCCCATTCAAGGCCTTCACCTGTTGTGCCAAGTCCATTTTCTAATGACTTCCTCCTCATTTGATCCAAGATGAATAGCACACATGCA  2280
         ||||||||||||||||||||||||||||||||||||||||||||||||||||||||||||||||||||||||||||||||||||||||||||||||||||||||||||||||||||||||
8638750  ACAGTGAGTCCAGGGCCAAAACCAAACAGCACACCCCATTCAAGGCCTTCACCTGTTGTGCCAAGTCCATTTTCTAATGACTTCCTCCTCATTTGATCCAAGATGAATAGCACACATGCA  8638869

2281     CTTGACATGTTACCATACTCGCTGAGCACATGCCTAGTAGCTTCCATTTTTTCAGGCTTCAAGCCTAACTTAGCCTCCACTTGGTCCAAAATTGCGGGTCCACCAGGGTGTGCAATCCAG  2400
         ||||||||||||||||||||||||||||||||||||||||||||||||||||||||||||||||||||||||||||||||||||||||||||||||||||||||||||||||||||||||
8638870  CTTGACATGTTACCATACTCGCTGAGCACATGCCTAGTAGCTTCCATTTTTTCAGGCTTCAAGCCTAACTTAGCCTCCACTTGGTCCAAAATTGCGGGTCCACCAGGGTGTGCAATCCAG  8638989

2401     AAGATAGAATTGTAATCGGAGATTCCCAAGGGTTGGAAGGCTTCAACCAAGGCCTTCTCAATATTCTTGGAGATGAGTCCAGGAACATCCTTGAGGAGATGGAAAGTGAGACCAACTTCG  2520
         ||||||||||||||||||||||||||||||||||||||||||||||||||||||||||||||||||||||||||||||||||||||||||||||||||||||||||||||||||||||||
8638990  AAGATAGAATTGTAATCGGAGATTCCCAAGGGTTGGAAGGCTTCAACCAAGGCCTTCTCAATATTCTTGGAGATGAGTCCAGGAACATCCTTGAGGAGATGGAAAGTGAGACCAACTTCG  8639109

2521     CGAAGGTGTCCATCAATAGCCCCTTCACTGTCTGGAAGGATTGTCTGGGCAGTCCAGACAAGCTGAAACAAAGGCTTTTCAACTGGTAAGGGGTCTGATCCAACAATGACAGCGGCTGCA  2640
         ||||||||||||||||||||||||||||||||||||||||||||||||||||||||||||||||||||||||||||||||||||||||||||||||||||||||||||||||||||||||
8639110  CGAAGGTGTCCATCAATAGCCCCTTCACTGTCTGGAAGGATTGTCTGGGCAGTCCAGACAAGCTGAAACAAAGGCTTTTCAACTGGTAAGGGGTCTGATCCAACAATGACAGCGGCTGCA  8639229

2641     CCATCTCCAAACAAGGCTTGACCCACAAGGCTATCAAGATGGGTGTCAGTTGGGCCACGGAATGTGACTGCGGTGATCTCAGAACAAACGACAAGCACGCGAGCACCCTTGTTGTTTTCA  2760
         ||||||||||||||||||||||||||||||||||||||||||||||||||||||||||||||||||||||||||||||||||||||||||||||||||||||||||||||||||||||||
8639230  CCATCTCCAAACAAGGCTTGACCCACAAGGCTATCAAGATGGGTGTCAGTTGGGCCACGGAATGTGACTGCGGTGATCTCAGAACAAACGACAAGCACGCGAGCACCCTTGTTGTTTTCA  8639349

2761     GCGAGGTCTTTGGCCAAACGAAGCACCGTGCCACCGGCAAAGCAGCCTTGTTGGTACATCATGTAACGCTTGACGGAGGGACGAAGGCCTAATAGTTTAGTGAGCTGATAATCAGCACCA  2880
         ||||||||||||||||||||||||||||||||||||||||||||||||||||||||||||||||||||||||||||||||||||||||||||||||||||||||||||||||||||||||
8639350  GCGAGGTCTTTGGCCAAACGAAGCACCGTGCCACCGGCAAAGCAGCCTTGTTGGTACATCATGTAACGCTTGACGGAGGGACGAAGGCCTAATAGTTTAGTGAGCTGATAATCAGCACCA  8639469

2881     GGCATGTCGACACCACTAGTGGTGCAAAAGATGAGATGGGTAATCTTGGACTTGGGTTGACCCCATTCCTTGATTGCCTTAGTTGCAGCCTCTTTTCCCAACTTTGGTACCTCCACAACC  3000
         ||||||||||||||||||||||||||||||||||||||||||||||||||||||||||||||||||||||||||||||||||||||||||||||||||||||||||||||||||||||||
8639470  GGCATGTCGACACCACTAGTGGTGCAAAAGATGAGATGGGTAATCTTGGACTTGGGTTGACCCCATTCCTTGATTGCCTTAGTTGCAGCCTCTTTTCCCAACTTTGGTACCTCCACAACC  8639589

3001     ACCATGTCTTGCCTTGCATCCAACGAAGGTGCCATATATGCACAAACACTGGGATTCTCCTTCAGGATCTCTTCGTTTAAGTACATGTATCGCTTCTTAATCATCGACTTATCACCTGAT  3120
         ||||||||||||||||||||||||||||||||||||||||||||||||||||||||||||||||||||||||||||||||||||||||||||||||||||||||||||||||||||||||
8639590  ACCATGTCTTGCCTTGCATCCAACGAAGGTGCCATATATGCACAAACACTGGGATTCTCCTTCAGGATCTCTTCGTTTAAGTACATGTATCGCTTCTTAATCATCGACTTATCACCTGAT  8639709

3121     CGAAATAATAAATTAAATTTCATTAATATCACTAATCAAATCAAAGGAATATGTTGAAAATAAGCAAAAACTTAAAGTTGGAATAAAATTTGGCAAAAACTAATAAGTTAGGGATAAAAA  3240
         ||||||||||||||||||||||||||||||||||||||||||||||||||||||||||||||||||||||||||||||||||||||||||||||||||||||||||||||||||||||||
8639710  CGAAATAATAAATTAAATTTCATTAATATCACTAATCAAATCAAAGGAATATGTTGAAAATAAGCAAAAACTTAAAGTTGGAATAAAATTTGGCAAAAACTAATAAGTTAGGGATAAAAA  8639829

3241     AAATATAATTATGTAACTAGTAAAGTGATGAAGGATAAAATTTGTAGGATTATTAAAAGTTGAGATAAAATGTCCAAAATTTAAAGATTAAGATAAAATTCGTCAAAAATTAAAAAATTA  3360
         ||||||||||||||||||||||||||||||||||||||||||||||||||||||||||||||||||||||||||||||||||||||||||||||||||||||||||||||||||||||||
8639830  AAATATAATTATGTAACTAGTAAAGTGATGAAGGATAAAATTTGTAGGATTATTAAAAGTTGAGATAAAATGTCCAAAATTTAAAGATTAAGATAAAATTCGTCAAAAATTAAAAAATTA  8639949
```

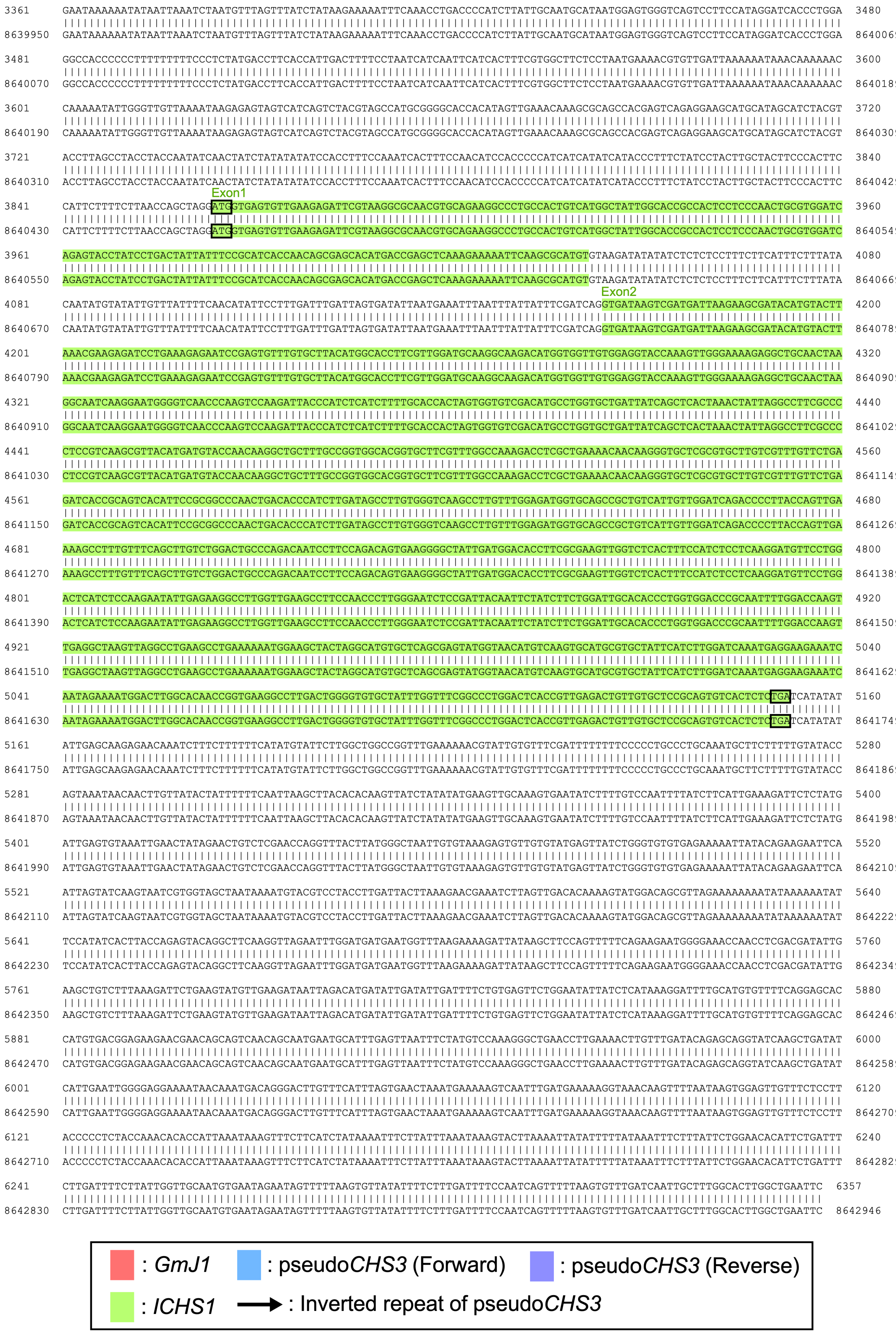

Supplemental Fig. 2. Nucleotide sequence comparison between the 'Enrei' genome (lower) and the *GmIRCHS*–*ICHS1* cluster (upper) registered in the DDBJ/EMBL/GenBank under the accession number AB264311. Vertical bars indicate identical nucleotides. Part of Exon1 of *GmJ1*, pseudo*CHS3* (forward), and pseudo*CHS3* (reverse) are shaded in light red, light blue, and light purple, respectively. Tail-to-tail-arranged inverted repeat of pseudo*CHS3* comprising *GmIRCHS* is indicated by black horizontal arrows. Start and stop codons of the *ICHS1* are enclosed in boxes. Two exons (Exon1 and Exon2) of *ICHS1* are shaded in light green.

(A)

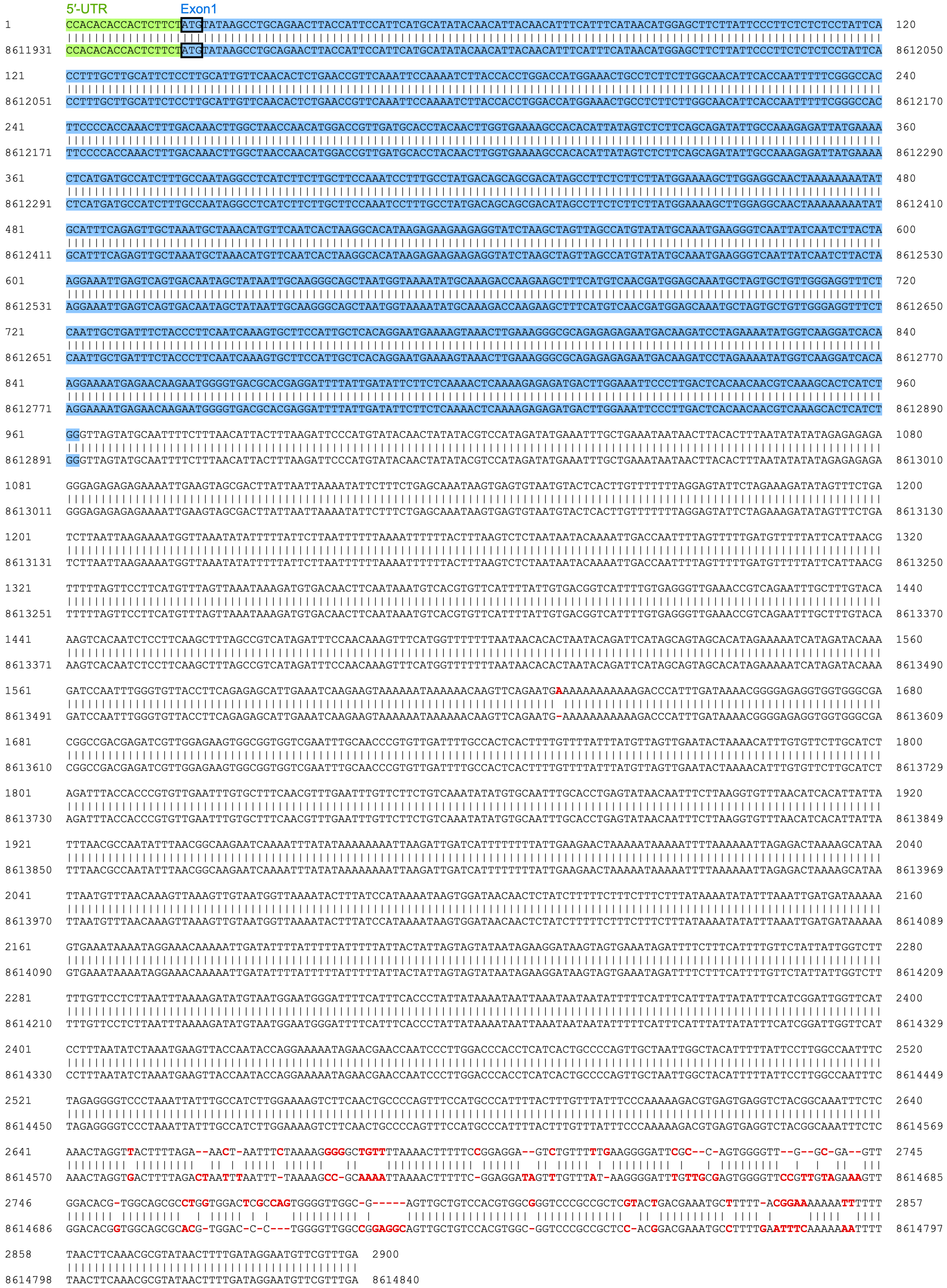

(B)

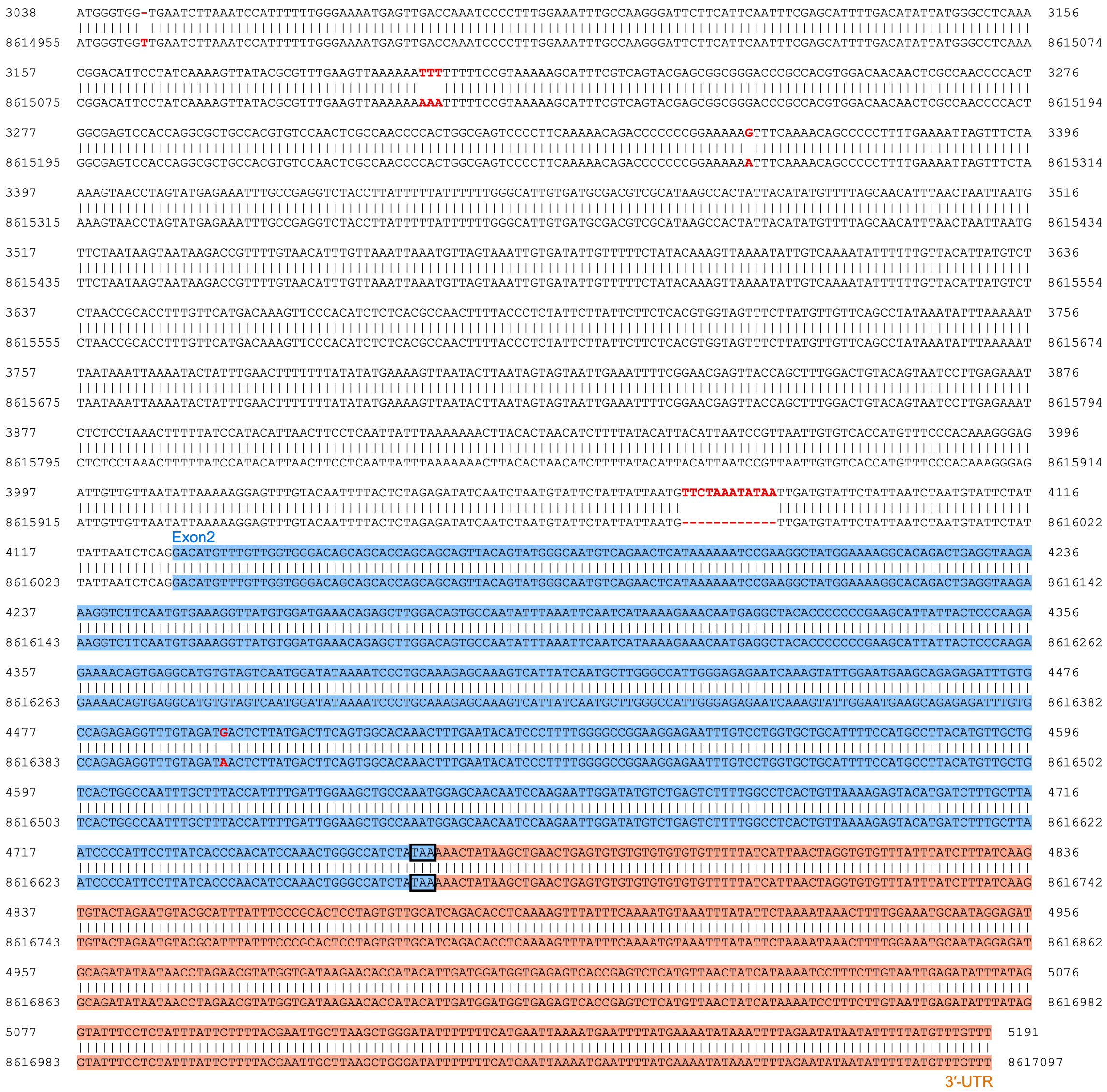

Supplemental Fig. 3. Nucleotide sequence comparison of a cytochrome P450 gene (*P450*) between the 'Wm82' genome (Glyma.08G109900) (upper) and the 'Enrei' genome at the approximately 8,610-kb position (lower). (A) 5′-portion of *P450* and (B) 3′-portion of *P450*. Vertical bars indicate identical nucleotides. Dashes (-) and nucleotides in red type denote gaps and different nucleotides, respectively. The 5′-UTR, two exons (Exon1 and Exon2), and 3′-UTR are shaded in light green, light blue and light orange, respectively. Start and stop codons are enclosed in boxes.

```
              5′-UTR                                                                  Exon1
1        ACACACAACCAAACCCTCTTGACCTTGTGATATCTCTCTCTTTTAATTTCTAGGATCAAACCAAAAAATGGTCTTAGAAGAGAGTGTAGTGCATGATGTGAGGCTATCCTCAGTTGGGCC  120
         ||||||||||||||||||||||||||||||||||||||||||||||||||||||||||||||||||||||||||||||||||||||||||||||||||||||||||||||||||||||||
8623430  ACACACAACCAAACCCTCTTGACCTTGTGATATCTCTCTCTTTTAATTTCTAGGATCAAACCAAAAAATGGTCTTAGAAGAGAGTGTAGTGCATGATGTGAGGCTATCCTCAGTTGGGCC  8623311

121      GGGCCGGGCCACCGGGTCGGACGTATTCCACAACCCGGGTGGCTTGGACTTGGCCATGAAGCTTCACTACCTTAGAGTGGTGTACTTCTTTGATAGTGAGGCTGCACAAGACCTAACCAT  240
         ||||||||||||||||||||||||||||||||||||||||||||||||||||||||||||||||||||||||||||||||||||||||||||||||||||||||||||||||||||||||
8623310  GGGCCGGGCCACCGGGTCGGACGTATTCCACAACCCGGGTGGCTTGGACTTGGCCATGAAGCTTCACTACCTTAGAGTGGTGTACTTCTTTGATAGTGAGGCTGCACAAGACCTAACCAT  8623191

241      CATGAAAATTAAGGATGGCATGTTCACTTTGTTCAACCATTACTTCATCACCTGTGGCCGGTTCCGGCGATCGGATTCCGGTCGACCCTTAATCAAGTGCAATGATTGTGGAGCAAGGTT  360
         ||||||||||||||||||||||||||||||||||||||||||||||||||||||||||||||||||||||||||||||||||||||||||||||||||||||||||||||||||||||||
8623190  CATGAAAATTAAGGATGGCATGTTCACTTTGTTCAACCATTACTTCATCACCTGTGGCCGGTTCCGGCGATCGGATTCCGGTCGACCCTTAATCAAGTGCAATGATTGTGGAGCAAGGTT  8623071

361      CATTGAGGCCAAGTGCAACAAAACCCTAGATGAGTGGCTAGCCATGAAGGATTGGCCCTTGTACAAGTTGCTCGTCTCTCACCAAGTCATTGGCCCAGAACTATCTTTTTCTCCTCCTGT  480
         ||||||||||||||||||||||||||||||||||||||||||||||||||||||||||||||||||||||||||||||||||||||||||||||||||||||||||||||||||||||||
8623070  CATTGAGGCCAAGTGCAACAAAACCCTAGATGAGTGGCTAGCCATGAAGGATTGGCCCTTGTACAAGTTGCTCGTCTCTCACCAAGTCATTGGCCCAGAACTATCTTTTTCTCCTCCTGT  8622951

481      TTTGTTCCAGGTGCTTAATAATTTCCCTAAACATTCTATATGATGCATCACACCTGACACACACATATTATTCATTTTTTTAAATATTACAAAGTACTACTTTCATACGATTTTTTTAGT  600
         ||||||||||||||||||||||||||||||||||||||||||||||||||||||||||||||||||||||||||||||||||||||||||||||||||||||||||||||||||||||||
8622950  TTTGTTCCAGGTGCTTAATAATTTCCCTAAACATTCTATATGATGCATCACACCTGACACACACATATTATTCATTTTTTTAAATATTACAAAGTACTACTTTCATACGATTTTTTTAGT  8622831

601      GTTATTTTTATCAGAATTTTTCATTTCTGTAATTTCCTCACTGTAAACCAAAGTACATTTATATTTTGATTAATTTACCAGATGAATGCCAGTGAAGAAATAGCTTAGCTTTTGCCTTCT  720
         ||||||||||||||||||||||||||||||||||||||||||||||||||||||||||||||||||||||||||||||||||||||||||||||||||||||||||||||||||||||||
8622830  GTTATTTTTATCAGAATTTTTCATTTCTGTAATTTCCTCACTGTAAACCAAAGTACATTTATATTTTGATTAATTTACCAGATGAATGCCAGTGAAGAAATAGCTTAGCTTTTGCCTTCT  8622711

721      TGACAAACAGAAAATTTGATATTGAAATTCAGTTATAAATACCTTTAAAGTGATTTTTATCAAACATATATAATAAAATAAAGTTAAAATTATTTTTTCACCATCCAAATATGATTTAAA  840
         ||||||||||||||||||||||||||||||||||||||||||||||||||||||||||||||||||||||||||||||||||||||||||||||||||||||||||||||||||||||||
8622710  TGACAAACAGAAAATTTGATATTGAAATTCAGTTATAAATACCTTTAAAGTGATTTTTATCAAACATATATAATAAAATAAAGTTAAAATTATTTTTTCACCATCCAAATATGATTTAAA  8622591

841      CTTTAGGTTTAATTACAGTTTTTACTCCAATTTTTTTTAATTTTTGACAAACTTTAACTTCAACTTTTGATTTAGTGCATTTTCTTCAACTTTTAAGAAAATTATAAATTTTATCTATTA  960
         ||||||||||||||||||||||||||||||||||||||||||||||||||||||||||||||||||||||||||||||||||||||||||||||||||||||||||||||||||||||||
8622590  CTTTAGGTTTAATTACAGTTTTTACTCCAATTTTTTTTAATTTTTGACAAACTTTAACTTCAACTTTTGATTTAGTGCATTTTCTTCAACTTTTAAGAAAATTATAAATTTTATCTATTA  8622471

961      ATAAGCACAAAAGTTAAGGATGAAATTTGTCAAAAAAAAATTAAGAGTAAAATTTGTAAAAAAAATTAAAAAGTTGGAGAAATTTTGTAAAAACAATAATAATAAAATAATAATAAGTTAAG  1080
         ||||||||||||||||||||||||||||||||||||||||||||||||||||||||||||||||||||||||||||||||||||||||||||||||||||||||||||||||||||||||
8622470  ATAAGCACAAAAGTTAAGGATGAAATTTGTCAAAAAAAAATTAAGAGTAAAATTTGTAAAAAAAATTAAAAAGTTGGAGAAATTTTGTAAAAACAATAATAATAAAATAATAATAAGTTAAG  8622351

1081     GATAAAAAGTGTAATTATGTTAGTAATAAATATTTTTTAATTTTTTAATCAATATGCTAAAGTTACTTGTTAATGATAAAAAAAAATATATATTATAAGTTTCTCTAGATACAAAATTGGT  1200
         ||||||||||||||||||||||||||||||||||||||||||||||||||||||||||||||||||||||||||||||||||||||||||||||||||||||||||||||||||||||||
8622350  GATAAAAAGTGTAATTATGTTAGTAATAAATATTTTTTAATTTTTTAATCAATATGCTAAAGTTACTTGTTAATGATAAAAAAAAATATATATTATAAGTTTCTCTAGATACAAAATTGGT  8622231

1201     ACTCCAAAGCTATTTTCCTTGGGTACTGACTGTTGTTGAGGACTGTTTTTTTTTTTTTATCTGTATTGATTTAACATTTCAAAAGATGATCTACTTTTTACGGGAAAAGAAAATGGCCAAA  1320
         ||||||||||||||||||||||||||||||||||||||||||||||||||||||||||||||||||||||||||||||||||||||||||||||||||||||||||||||||||||||||
8622230  ACTCCAAAGCTATTTTCCTTGGGTACTGACTGTTGTTGAGGACTGTTTTTTTTTTTTTATCTGTATTGATTTAACATTTCAAAAGATGATCTACTTTTTACGGGAAAAGAAAATGGCCAAA  8622111

1321     ATTCTTTTCAATATTTCTCTCTTTCTCCCTCTCACCCAAAATGGCCAAACACATTATAAATACAGCAGTAATTATAAATACTGTAGTCAATGCCATAAAAAGAGTAGCTACTAGCTAGCT  1440
         ||||||||||||||||||||||||||||||||||||||||||||||||||||||||||||||||||||||||||||||||||||||||||||||||||||||||||||||||||||||||
8622110  ATTCTTTTCAATATTTCTCTCTTTCTCCCTCTCACCCAAAATGGCCAAACACATTATAAATACAGCAGTAATTATAAATACTGTAGTCAATGCCATAAAAAGAGTAGCTACTAGCTAGCT  8621991

1441     GGGGTTTAGTCCTGCTACACTAGTGAGTGGTGACTACAGCTGTGCAACACGTGTTTGTCTGACACTTTTAAGTTTTACCGAATCTTTGATACATTAATAAAATGGAGAAAGAAAAAAAAT  1560
         ||||||||||||||||||||||||||||||||||||||||||||||||||||||||||||||||||||||||||||||||||||||||||||||||||||||||||||||||||||||||
8621990  GGGGTTTAGTCCTGCTACACTAGTGAGTGGTGACTACAGCTGTGCAACACGTGTTTGTCTGACACTTTTAAGTTTTACCGAATCTTTGATACATTAATAAAATGGAGAAAGAAAAAAAAT  8621871

1561     ATATTTGAAATTTAAATAAGAACATTTTAAAATCATATTTCTCTCTGTATTTTCCATTTCCTAAAAAATAGTTATAACTAATTATTAAAAAATTAACAGCTAAACTTAAATATATGACTA  1680
         ||||||||||||||||||||||||||||||||||||||||||||||||||||||||||||||||||||||||||||||||||||||||||||||||||||||||||||||||||||||||
8621870  ATATTTGAAATTTAAATAAGAACATTTTAAAATCATATTTCTCTCTGTATTTTCCATTTCCTAAAAAATAGTTATAACTAATTATTAAAAAATTAACAGCTAAACTTAAATATATGACTA  8621751

1681     TATACAGAAAACAAATCACACAATTGTTAAAATTTATTCTCAACTAGTATACATTTTTAATGAACGATTAAAATATTTCCTCATTTAATGTGCCCCTCTGCACAATATAGTACAATAAAA  1800
         ||||||||||||||||||||||||||||||||||||||||||||||||||||||||||||||||||||||||||||||||||||||||||||||||||||||||||||||||||||||||
8621750  TATACAGAAAACAAATCACACAATTGTTAAAATTTATTCTCAACTAGTATACATTTTTAATGAACGATTAAAATATTTCCTCATTTAATGTGCCCCTCTGCACAATATAGTACAATAAAA  8621631

1801     CGTGCGAAAGGACCCTAACACTCTTTATCATGTCCTGGTTTGGTTGAAACAGTGACCTGTTAATTAGTCGCATGCTTTTTCTGGTAGAGAAGTTAATAGCATCCTATCTTCATAAGTTAC  1920
         ||||||||||||||||||||||||||||||||||||||||||||||||||||||||||||||||||||||||||||||||||||||||||||||||||||||||||||||||||||||||
8621630  CGTGCGAAAGGACCCTAACACTCTTTATCATGTCCTGGTTTGGTTGAAACAGTGACCTGTTAATTAGTCGCATGCTTTTTCTGGTAGAGAAGTTAATAGCATCCTATCTTCATAAGTTAC  8621511

1921     CCTTACATTAATACAAATAGTATTATATGGTGGACATTCCTTTCATAAGAGAGATAAGGTAGATCTTAACTATTCAACAAAAAAAGAAAAAAGGTGCATCCAAACATATTTGAATATTTC  2040
         ||||||||||||||||||||||||||||||||||||||||||||||||||||||||||||||||||||||||||||||||||||||||||||||||||||||||||||||||||||||||
8621510  CCTTACATTAATACAAATAGTATTATATGGTGGACATTCCTTTCATAAGAGAGATAAGGTAGATCTTAACTATTCAACAAAAAAAGAAAAAAGGTGCATCCAAACATATTTGAATATTTC  8621391

2041     TGTAGACCGTAAGCTAAAAGCCCTCGGTATCATTAATAAGGATTCCTTTATACTGTAAAATACATCATTACTTGTTTTTTATAAAAATAAAATAAAATCCGAGGTAATATTTTGATTTTA  2160
         ||||||||||||||||||||||||||||||||||||||||||||||||||||||||||||||||||||||||||||||||||||||||||||||||||||||||||||||||||||||||
8621390  TGTAGACCGTAAGCTAAAAGCCCTCGGTATCATTAATAAGGATTCCTTTATACTGTAAAATACATCATTACTTGTTTTTTATAAAAATAAAATAAAATCCGAGGTAATATTTTGATTTTA  8621271

2161     AGTAATGCTAATCTTCTTATAGTTTTGTTAACACTTTATAAATAAATATTATATGAAAAAATAAATGCAATTAATGTCTTATAAAAAATTACTTAATATTTAATAGTAGTACTACATATT  2280
         ||||||||||||||||||||||||||||||||||||||||||||||||||||||||||||||||||||||||||||||||||||||||||||||||||||||||||||||||||||||||
8621270  AGTAATGCTAATCTTCTTATAGTTTTGTTAACACTTTATAAATAAATATTATATGAAAAAATAAATGCAATTAATGTCTTATAAAAAATTACTTAATATTTAATAGTAGTACTACATATT  8621151
```

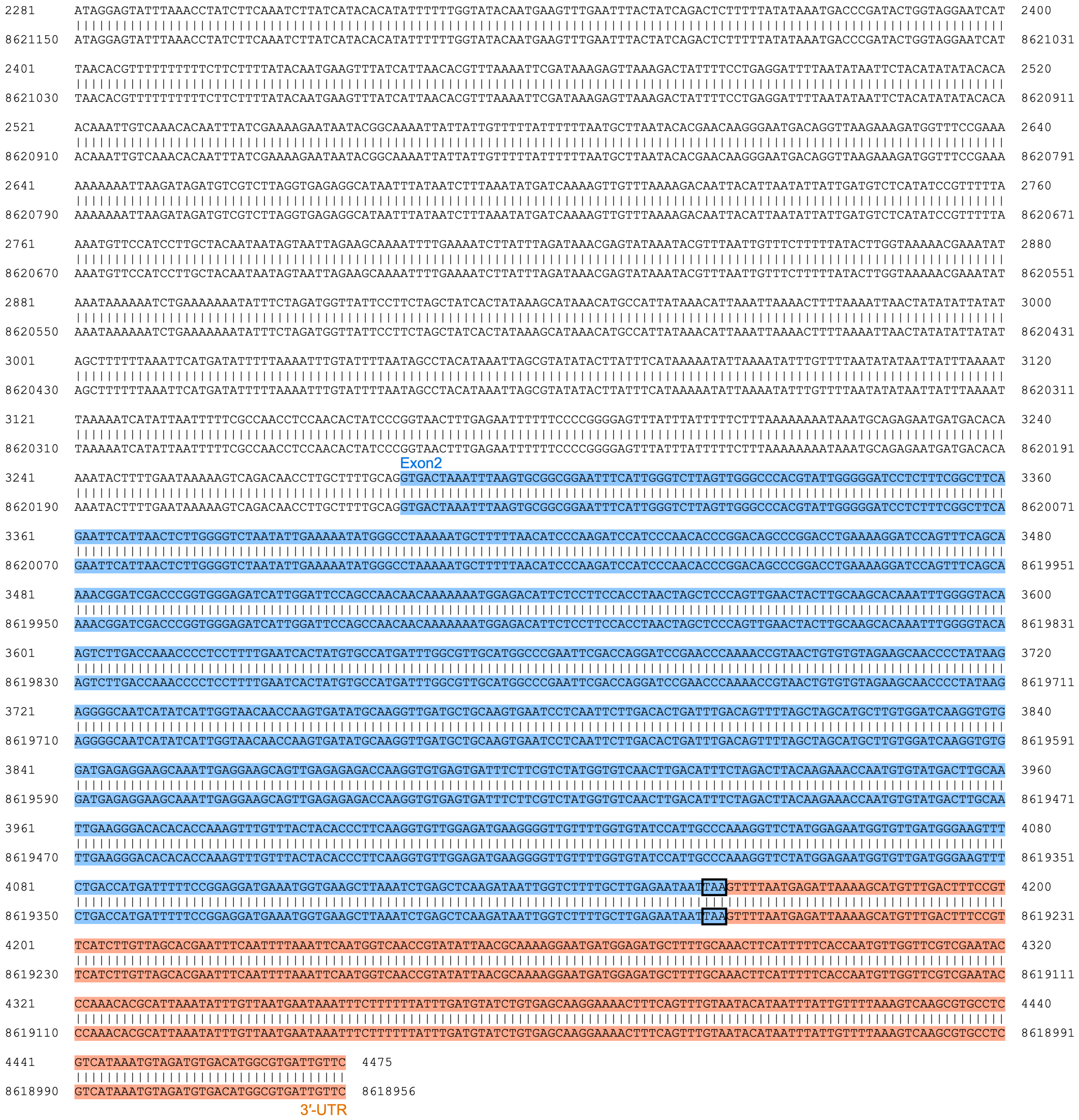

Supplemental Fig. 4. Nucleotide sequence comparison of a Transferase gene (*Transferase*) between the 'Wm82' genome (Glyma.08G110000) (upper) and the 'Enrei' genome at the approximately 8,620-kb position (lower). Vertical bars denote identical nucleotides. 5′-UTR, two exons (Exon1 and Exon2), and 3′-UTR are shaded in light green, light blue and light orange, respectively. Start and stop codons are enclosed in boxes.

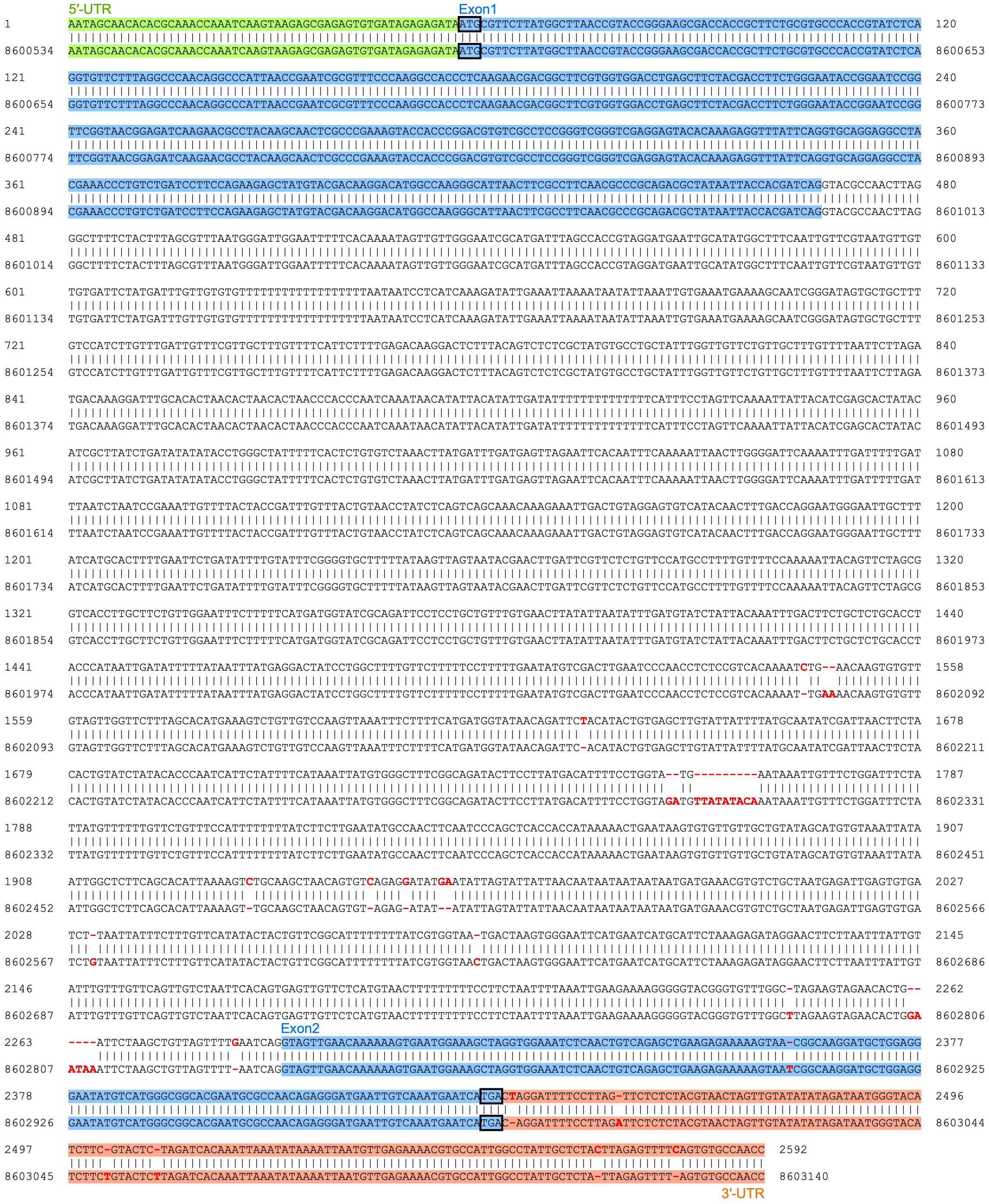

Supplemental Fig. 5. Nucleotide sequence comparison of a DnaJ gene (*GmJ1*) between the 'Wm82' genome (Glyma.08G109700) (upper) and the 'Enrei' genome at the approximately 8,600-kb position (lower). Vertical bars denote identical nucleotides. Dashes (-) and nucleotides in red type indicate gaps and different nucleotides, respectively. The 5′-UTR, two exons (Exon1 and Exon2), and 3′-UTR are shaded in light green, light blue and light orange, respectively. Start and stop codons are enclosed in boxes.

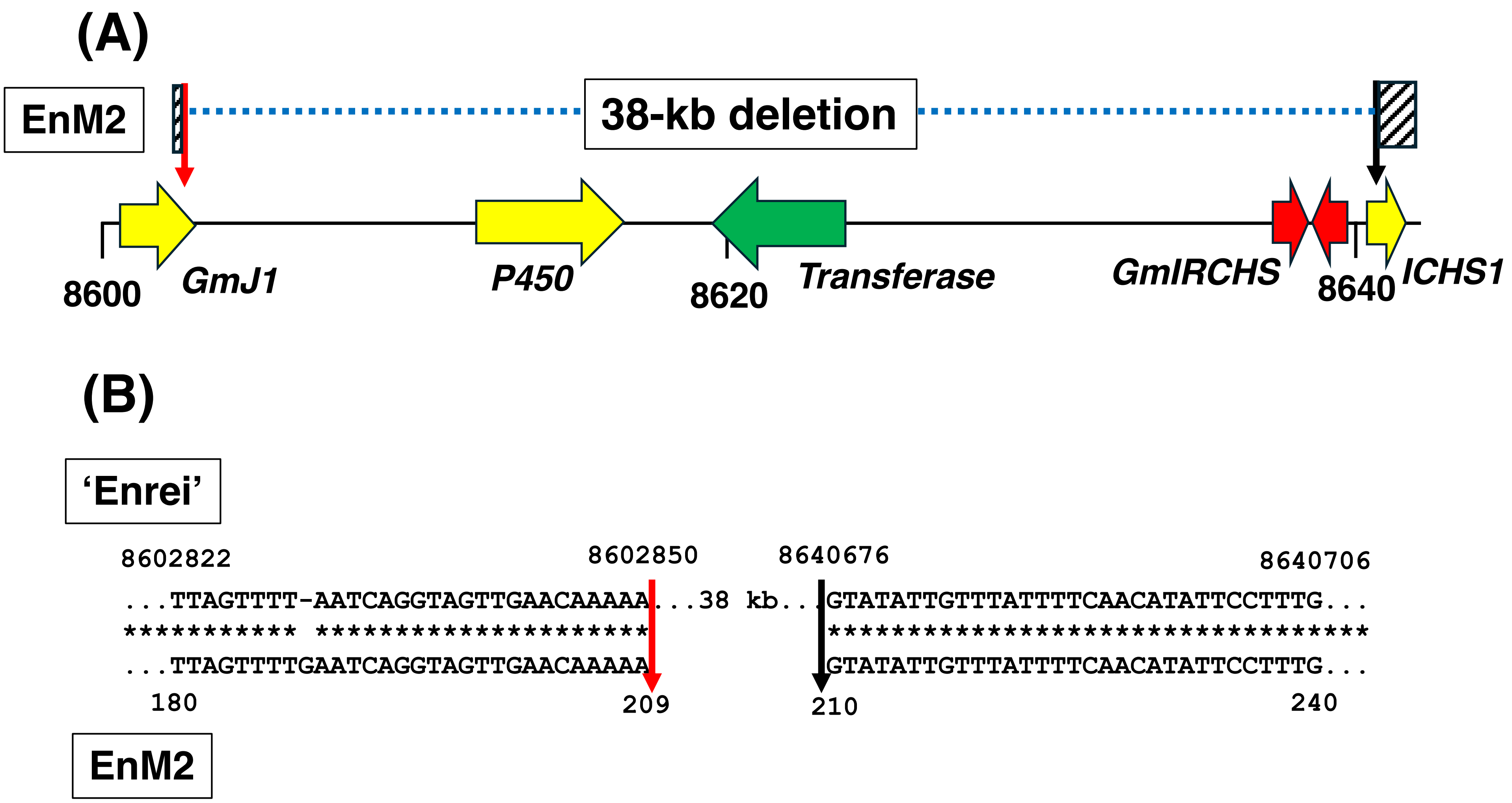

Supplemental Fig. 6. The 38-kb deletion region identified in EnM2. (A) Locations of the *GmIRCHS-ICHS1* cluster and three genes (*GmJ1*, *P450*, and *Transferase*) in the 'Enrei' genome. Hatched boxes indicate the region amplified by inverse PCR. Positions of the 5′- and 3′-ends of the deletion region are denoted by vertical red and black arrows, respectively.
(B) Comparison between the 'Enrei' genome sequence (upper) and the nucleotide sequence in EnM2 (AB822566) corresponding to the DNA region indicated by the hatched boxes in panel A (lower). Asterisks indicate identical nucleotides. Numbers of genome positions of 'Enrei' chr08 and nucleotide numbers of the sequence in EnM2 are also indicated.

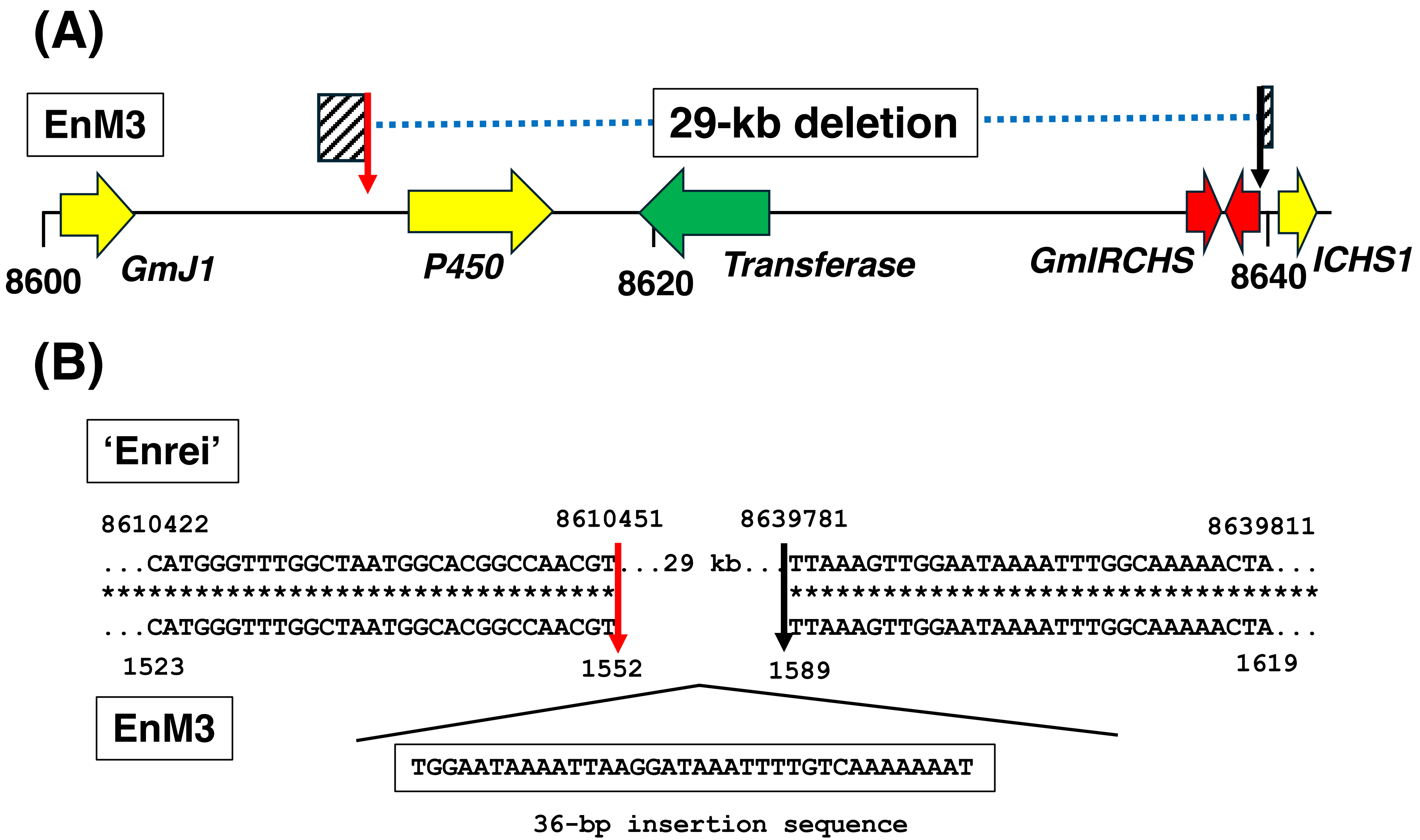

Supplemental Fig. 7. The 29-kb deletion region identified in EnM3. (A) Locations of the *GmIRCHS-ICHS1* cluster and three genes (*GmJ1*, *P450*, and *Transferase*) in the 'Enrei' genome. Hatched boxes indicate the region amplified by inverse PCR. Positions of the 5′- and 3′-ends of the deletion region are denoted by vertical red and black arrows, respectively.
(B) Comparison between the 'Enrei' genome sequence (upper) and the nucleotide sequence in EnM3 (AB822567) corresponding to the DNA region indicated by the hatched boxes in panel A (lower). Asterisks indicate identical nucleotides. Numbers of genome positions of 'Enrei' chr08 and nucleotide numbers of the sequence in EnM3 are also indicated. A 36-bp insertion sequence (Senda *et al*. 2013) at a deletion point is enclosed in a box.

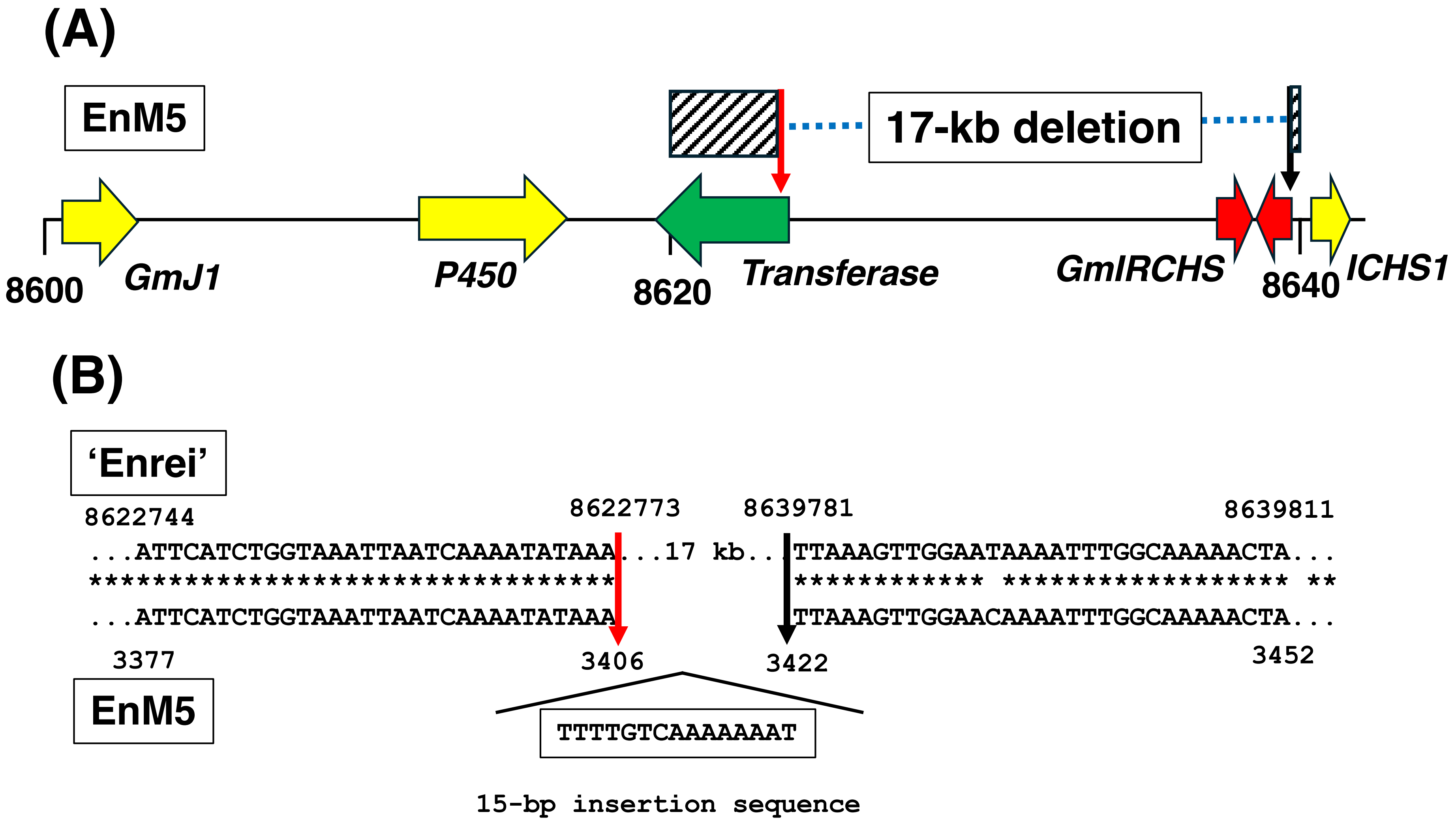

Supplemental Fig. 8. The 17-kb deletion region identified in EnM5. (A) Locations of the *GmIRCHS-ICHS1* cluster and three genes (*GmJ1*, *P450*, and *Transferase*) in the 'Enrei' genome. Hatched boxes indicate the region amplified by inverse PCR. Positions of the 5′- and 3′-ends of the deletion region are denoted by vertical red and black arrows, respectively.
(B) Comparison between the 'Enrei' genome sequence (upper) and the nucleotide sequence in EnM5 (AB822569) corresponding to the DNA region indicated by the hatched boxes in panel A (lower). Asterisks indicate identical nucleotides. Numbers of genome positions of 'Enrei' chr08 and nucleotide numbers of the sequence in EnM5 are also indicated. A 15-bp insertion sequence (Senda *et al*. 2013) at a deletion point is enclosed in a box.

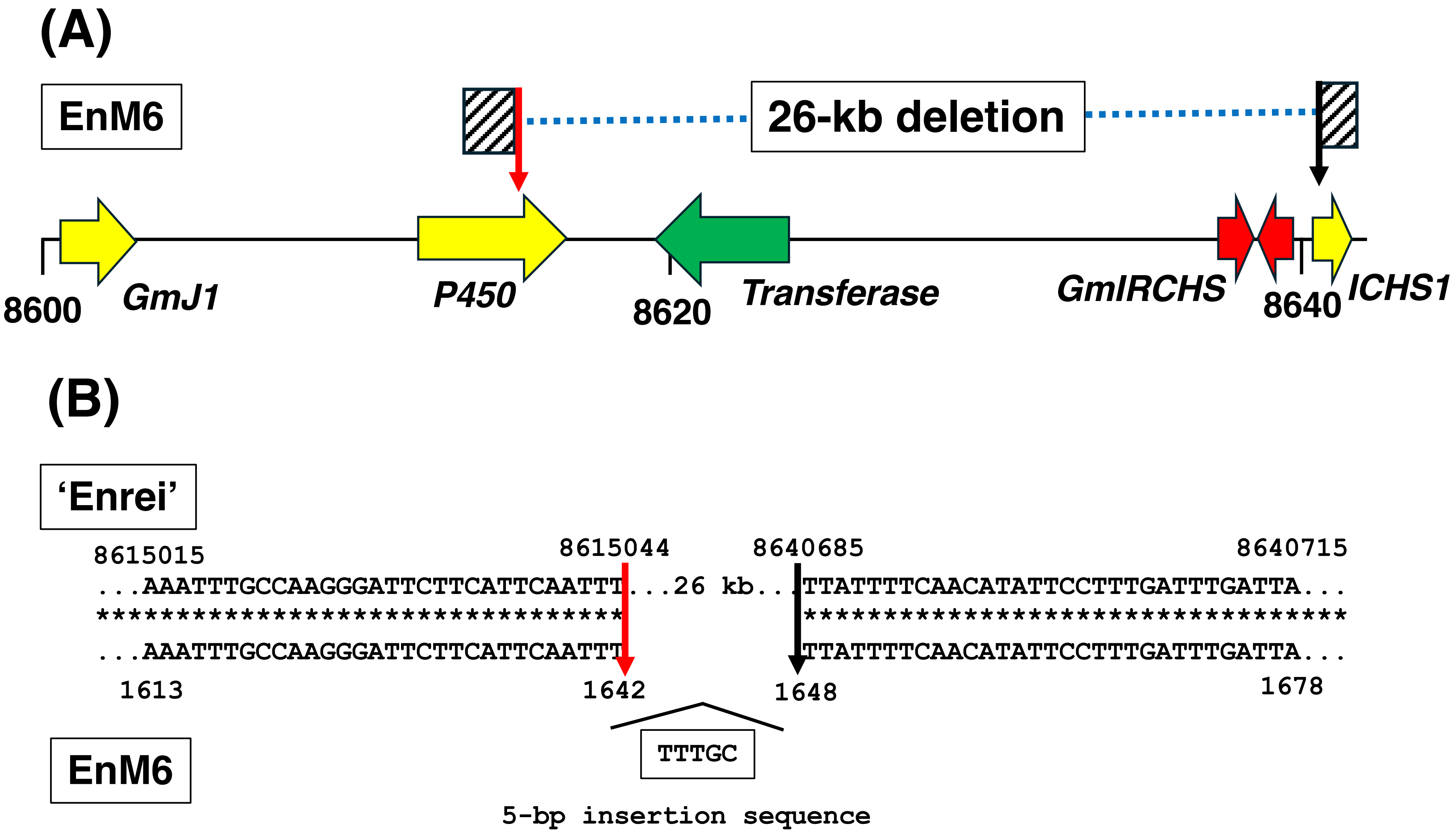

Supplemental Fig. 9. The 26-kb deletion region identified in EnM6. (A) Locations of the *GmIRCHS-ICHS1* cluster and three genes (*GmJ1*, *P450*, and *Transferase*) in the 'Enrei' genome. Hatched boxes indicate the region amplified by inverse PCR. Positions of the 5′- and 3′-ends of the deletion region are denoted by vertical red and black arrows, respectively.
(B) Comparison between the 'Enrei' genome sequence (upper) and the nucleotide sequence in EnM6 (AB822570) corresponding to the DNA region indicated by the hatched boxes in panel A (lower). Asterisks indicate identical nucleotides. Numbers of genome positions of 'Enrei' chr08 and nucleotide numbers of the sequence in EnM6 are also indicated. A 5-bp insertion sequence (Senda *et al*. 2013) at a deletion point is enclosed in a box.

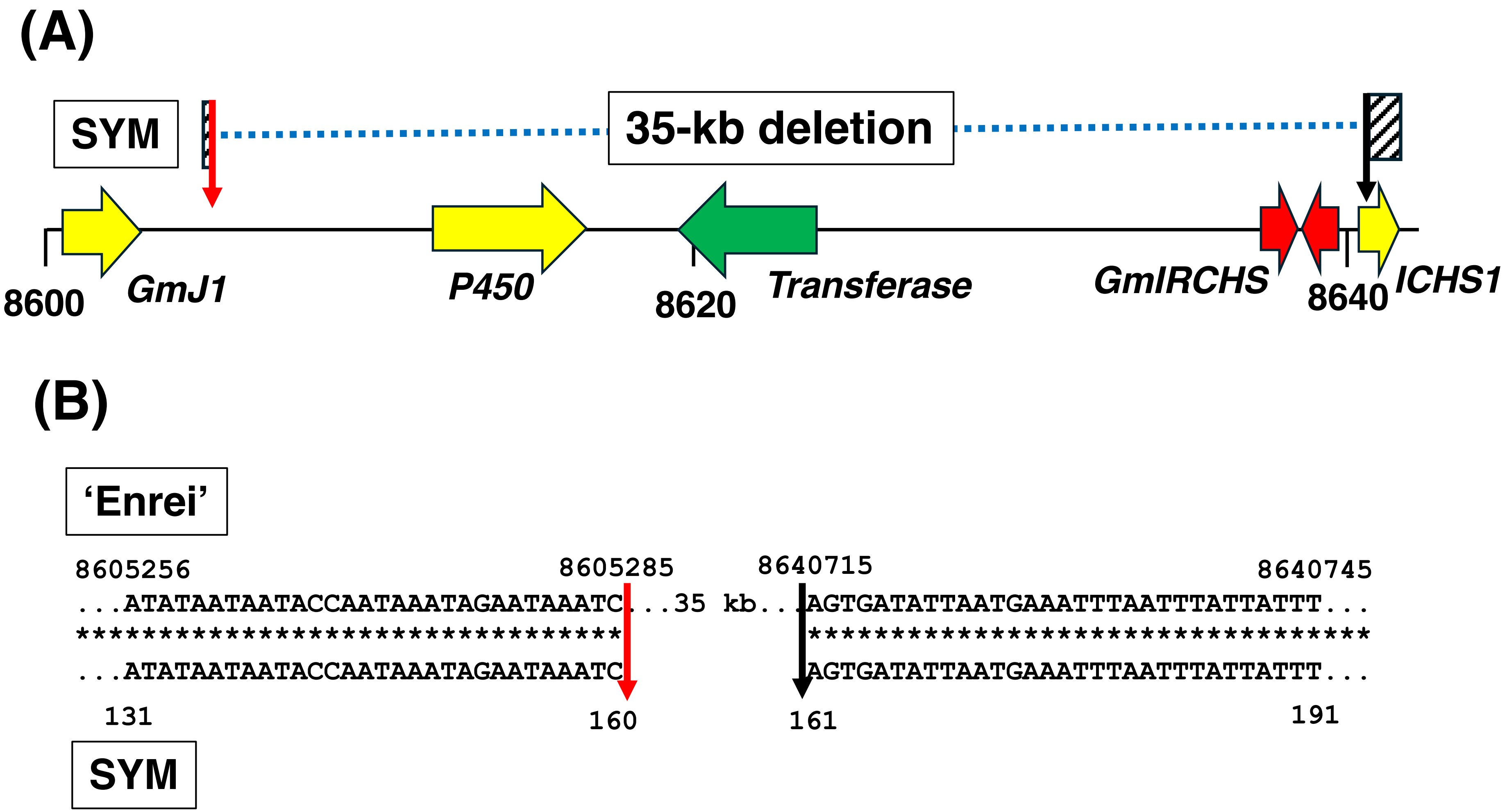

Supplemental Fig. 10. The 35-kb deletion region identified in SYM. (A) Locations of the *GmIRCHS-ICHS1* cluster and three genes (*GmJ1*, *P450*, and *Transferase*) in the ‘Enrei’ genome. The size of deletion region in SYM was estimated based on the assumption that the genomic structure of ‘Suzuyutaka’ is identical to that of ‘Enrei’ at this locus. Hatched boxes indicate the region amplified by PCR. Positions of the 5′- and 3′-ends of the deletion region are denoted by vertical red and black arrows, respectively. (B) Comparison between the ‘Enrei’ genome sequence (upper) and the nucleotide sequence in SYM (LC871491) corresponding to the DNA region indicated by the hatched boxes in panel A (lower). Asterisks indicate identical nucleotides. Numbers of genome positions of Enrei chr08 and nucleotide numbers of the sequence in SYM are also indicated.
